# An Amphipathic Alpha-Helix in the Prodomain of Cocaine and Amphetamine Regulated Transcript Peptide Precursor Serves as Its Sorting Signal to the Regulated Secretory Pathway

**DOI:** 10.1371/journal.pone.0059695

**Published:** 2013-03-19

**Authors:** Elías H. Blanco, Carlos F. Lagos, María Estela Andrés, Katia Gysling

**Affiliations:** 1 Millennium Science Nucleus in Stress and Addiction, Department of Cell and Molecular Biology, Faculty of Biological Sciences, Pontificia Universidad Católica de Chile, Santiago, Chile; 2 Department of Pharmacy, Faculty of Chemistry, Pontificia Universidad Católica de Chile, Santiago, Chile; University of Rome, Italy

## Abstract

Cocaine and Amphetamine Regulated Transcript (CART) peptides are anorexigenic neuropeptides. The L34F mutation in human CART peptide precursor (proCART) has been linked to obesity (Yanik et al. Endocrinology 147: 39, 2006). Decrease in CART peptide levels in individuals carrying the L34F mutation was attributed to proCART subcellular missorting. We studied proCART features required to enter the regulated secretory pathway. The subcellular localization and the secretion mode of monomeric EGFP fused to the full-length or truncated forms of human proCART transiently transfected in PC12 cells were analyzed. Our results showed that the N-terminal 1–41 fragment of proCART was necessary and sufficient to sort proCART to the regulated secretory pathway. *In silico* modeling predicted an alpha-helix structure located between residues 24–37 of proCART. Helical wheel projection of proCART alpha-helix showed an amphipathic configuration. The L34F mutation does not modify the amphipathicity of proCART alpha-helix and consistently proCART_L34F_ was efficiently sorted to the regulated secretory pathway. However, four additional mutations to proCART_L34F_ that reduced its alpha-helix amphipathicity resulted in the missorting of the mutated proCART toward the constitutive secretory pathway. These findings show that an amphipathic alpha-helix is a key cis-structure for the proCART sorting mechanism. In addition, our results indicate that the association between L34F mutation and obesity is not explained by proCART missorting.

## Introduction

Cocaine and Amphetamine Regulated Transcript (CART) was identified as an mRNA that increases its level in rat striatum after binge acute doses of cocaine or amphetamine [1]. Bioactive CART neuropeptides have potent anxiogenic and anorexigenic effects [2], [3]. Central injection of CART_42–89_ neuropeptide in the rodent brain increases anxiogenic behavior [2] and suppresses appetite [3], [4]. Moreover, central injection of CART_42–89_ neuropeptide inhibits the increase in appetite triggered by neuropeptide Y (NPY), a potent orexigenic neuropeptide [3], [5].

Human CART mRNA encodes a polypeptide of 116 amino acids including a signal peptide of 27 residues in its amino-terminal necessary for accessing the secretory pathway [6]. The CART neuropeptide precursor (proCART) of 89 residues is sorted and stored in secretory granules, a hallmark of the regulated secretory pathway [7]. Electron microscopy studies show CART peptide-like immunoreactivity in large dense core vesicles of nucleus accumbens neurons [8], [9]. proCART is processed by prohormone convertase 1/3 (PC1/3) and prohormone convertase 2 (PC2) [10]. Both, PC1/3 and PC2 enter the regulated secretory pathway and are stored in secretory granules [11]. The processing of proCART yields two bioactive CART neuropeptides, CART_42–89_ and CART_49–89_ [12], [13]. Therefore, the sorting of proCART into secretory granules is essential for its bioactivation. However, the mechanism by which proCART is sorted toward the secretory granules remains unknown.

The importance of proCART subcellular sorting was reinforced with the finding that a missense mutation that changes the leucine residue 34 to phenylalanine of proCART (proCART_L34F_) was associated with the occurrence of obesity in members of an Italian family carrying this mutation [14]. Family members with proCART_L34F_ show an obese phenotype and a dramatic decrease in serum CART peptide levels [15]. Besides, studies with heterologous expression of proCART in AtT20 cells led to the suggestion that the L34F mutation decreases the sorting of proCART towards the regulated secretory pathway increasing its constitutive secretion [15]. Therefore, it was proposed that the missorting of proCART L34F could explain the obese phenotype in this family.

In order to identify the proCART sorting domain and the effect of the L34F mutation in proCART sorting, we studied the subcellular localization of the full-length or truncated forms of human proCART fused to monomeric EGFP (EGFPm) expressed in PC12 cells. Subcellular localization of CART-EGFPm fusion proteins were compared with specific subcellular markers using confocal microscopy and quantitative analysis. In addition, secretion assays were performed measuring the sensitivity to barium-induced secretion of CART-EGFPm fusion proteins transfected PC12 cells.

We found that the N-terminal 1–41 fragment of proCART is necessary and sufficient to sort EGFPm into the regulated secretory pathway. *In silico* modeling showed that the N-terminal fragment of proCART contains an amphipathic alpha-helix. When the amphipathicity of the alpha-helix is reduced, proCART is missorted from the regulated secretory pathway. Surprisingly, we observed that proCART_L34F_ is readily sorted into the regulated secretory pathway.

## Materials and Methods

### Cloning and mutagenesis

In order to decrease the dimerization capacity of EGFP [16], we used the monomeric form of EGFP (EGFPm). This was achieved by mutating alanine 206 by a lysine (A206K) in the mammalian expression vector pEGFP-N3 (Clontech) as described [16]. EGFP_A206K_ and subsequent mutations were done by the PCR-driven overlap extension protocol [17]. Different fragments of proCART (including the 27 amino acid of its signal peptide, SP) were obtained by PCR using specific primers incorporating an *XhoI* restriction site followed by a Kozak translation initiation consensus sequence (GCCACC-ATG) at the 5′ end, and a *BamHI* restriction endonuclease site at the 3′ end. The human proCART cDNA, used as a template, was a kind gift of Dr. Patrick Keller [18]. The amplified fragments were purified, digested with *XhoI* and *BamHI* and subcloned in frame into the same sites of the expression vector for pEGFP (A206K)-N3 with the sequence flanked by *XhoI* and *BamHI* restriction sites. All fusion proteins were verified by restriction analysis and sequencing. In addition, adequate expression of each fusion protein was confirmed by western blot of total cell lysates.

### Cell culture and transfections

PC12 cells (ATCC, N° CRL-1721) were maintained in DMEM (Gibco), supplemented with 10% horse serum (Hyclone) and 5% fetal bovine serum (Gibco), and 100 IU/mL penicillin and 100 μg/mL streptomycin, at 37°C in an incubator with 10% CO_2_. In the last passage before transfection, PC12 cells were grown in tissue culture dishes treated with poly-L-lysine (50 µg/mL, Sigma). PC12 cells were transfected with Lipofectamine 2000 reagent (Invitrogen). For immunofluorescence studies, 0.20 µg of each plasmid were transfected in 24-well plates with a density of 10^5^ cells per well. Forty-eight hours after transfection, cells were fixed with 4% paraformaldehyde. For immunoblotting studies, 2 µg of each plasmid were transfected in 6-well plates with a density of 10^6^ cells per well. Forty-eight hours after transfection cells were harvested. For secretion assays, 10 µg of each plasmid were transfected in 100 mm dishes with a density of 6×10^6^ cells. Forty-eight hours after transfection, cells were incubated in fresh media to perform the secretion assays.

### Fluorescence immunocytochemistry

PC12 cells transfected with the different CART-EGFPm fusion proteins were cultured on poly-L-lysine coated glass coverslips and fixed for 30 minutes at room temperature with 4% paraformaldehyde in phosphate buffered saline (PBS), pH 7.4. Fixed PC12 cells were permeabilized with 0.2% Triton ×100/2.5% BSA for 30 min at room temperature. Cells were then incubated with rabbit polyclonal anti-secretogranin II (SgII, Abcam) 1∶100 as marker of secretory granules. Thereafter, cells were washed and incubated with Cy3 donkey anti-rabbit (1∶200, Jackson) as second antibody. Cells were washed and mounted with DAKO mounting media.

### Western blot

PC12 cells transfected with the fusion proteins were lysed in RIPA lysis buffer (Millipore) supplemented with a protease inhibitor cocktail (Complete Mini, Roche) during 15 minutes at 4°C. Samples were centrifuged at 14,000 r.p.m. for 15 minutes at 4°C and supernatant were collected. Proteins were separated by SDS-PAGE on 12% polyacrylamide gels and transferred onto nitrocellulose membrane (Trans-blot, BioRad). Membranes were blocked with a buffer containing 5% nonfat dry milk in 0.05% Tween-20 PBS, for 30 minutes. Thereafter, membranes were incubated for 48 hours with a monoclonal mouse anti-GFP Santa Cruz, sc-9996) 1∶1000 and washed 3 times for 15 minutes with 0.05% Tween-20 in PBS. Membranes were subsequently incubated for 1 hour with an anti-rabbit horseradish peroxidase conjugate secondary antibody (Jackson) 1∶5000 in 0.05% Tween-20 in PBS. Immunoreactive bands were detected by chemiluminescence (Supersignal West Pico, Pierce). For SgII western blot a polyclonal rabbit anti-SgII (1∶500, Abcam) was used.

### Confocal microscopy and Colocalization Analysis

Immunofluorescent images were obtained with a Fluoview 1000 confocal microscope (Olympus) and Flouview v6.0 software. Images were obtained with a 100× objective (N.A 1.4 oil) and using a sequential mode of laser scanning with 26 slices per cell (Z-step of 80 nm). In these conditions, each pixel corresponds to 38 nm. Images were processed in the IMAGE J software. Deconvolution and colocalization analysis were made with “Iterative Deconvolve 3D and “JaCoP” plugins [19].

Pearson's correlation coefficient [20] was the method used to analyze the subcellular colocalization for the different fusion proteins. Twenty-six z-planes obtained for each cell in at least three independent experiments were processed for each data. A total of 8 cells (208 images) for SgII-ir colocalization were analyzed for each fusion protein. In order to compare the Pearson's coefficient, values were analyzed with one-way ANOVA followed by Dunnet pos-hoc test with GraphPad Prism 5 software.

### Secretion assays

Secretion assays were performed essentially as described in Blanco et al. [21]. Transiently transfected PC12 cells were washed with basal secretion medium (150 mM NaCl, 5 mM KCl, 2 mM CaCl_2_, 10 mM HEPES pH 7.4) and exposed to this basal medium for 30 minutes. Extracellular media were collected (Basal sample) and immediately replaced by stimulus secretion medium (2 mM BaCl instead of 2 mM CaCl_2_) for 30 minutes. After that time, extracellular media were again collected (Stimulus sample). Basal secretion was also determined in the incubation media of the first 48 hs post-transfection. All extracellular media were cleared by centrifugation (5 minutes, 1000 rpm, 4°C), and concentrated using reverse phase Sep-Pak C-18-E columns (Strata, Phenomenex). Eluates were lyophilized and analyzed by western blot.

Quantitative analysis of basal secretion, plotted as percentage, was performed comparing the amount of each fusion protein present in the extracellular media with the respective cell lysate content. Densitometric profiles were obtained with ImageJ software.

### *In silico* Molecular Modeling

The molecular model of proCART was constructed using Modeller [22] as implemented in the Protein Modeling module of Discovery Studio 2.1 (Accelrys Inc., San Diego, CA). The sequence of mature proCART (Uniprot entry Q16568 residues) was retrieved from the Uniprot database [23]. Secondary structure prediction was performed using PCI-SS [24] and further analyzed with the NPS@ consensus secondary structure prediction program that includes 8 secondary structure prediction algorithms [25]. The C-terminal domain of proCART solved by NMR (PDB id 1HY9, residues 48-89) was used as the starting model [26]. Threading for the N-terminal region was performed using Phyre2 server [27]. A model obtained from fold fragments was retrieved and aligned, and used as template to generate a full wt proCART model. A total of 100 models were constructed and the best model according to Modeller internal score was subjected to a molecular minimization protocol using the CHARMM 22 force-field [28]. The protocol consisted of 5,000 steps of steepest descent method, followed by 10,000 steps of conjugate gradient method to reach a final root-mean square (RMS) gradient of 0.001 kcal/mol/Å. The overall quality of the final model was assessed by Ramachandran plot analysis using the RAMPAGE server [29] and Profiles-3D analysis [30]. Additional quality model assessments were performed using the ProSA-web [31], QProt [32] and SAVES server (http://nihserver.mbi.ucla.edu/). APBS software was used to calculate the spatial distribution of electrostatic potential on protein atoms using a two-dielectric implicit solvent model and the finite difference method to solve the Poisson-Boltzmann Equation. The dielectric constant used for protein was 2 and 80 for the solvent [33].

Helical wheel projections were carried out with the EMBOSS (European Molecular Biology Open Software Suite) software package [34]. CART amino acidic sequences used were: *Homo sapiens* (NCBI NP_004282.1), *Xenopus leavis* (NCBI NP_001087565.1), *Rattus novergicus* (NCBI NP_058806.1), *Mus musculus* variant 1 (NCBI NP_038760.3), *Mus musculus* variant 2 (NCBI NP_001074962.1), *Bos taurus* (NCBI NP_001007821.1), *Sus scrofa* (NCBI NP_001093395.1), *Macaca mulata* (NCBI NP_001252806.1), *Salmo salar* (NCBI NP_001140152.1) and *Danio rerio* (NCBI NP_001017570.1). To determine proCART amino acidic sequence, the Signal Peptides of pre-proCART sequences (obtained from NBCI database) were determined using “SignalP 4.0 server” web-software [35]. For multiple proCART sequence alignment, we used “ClustalW2” web-software [36].

## Results

### The 1–41 N-terminal fragment of proCART is necessary and sufficient to enter the regulated secretory pathway

**Figure 1:**
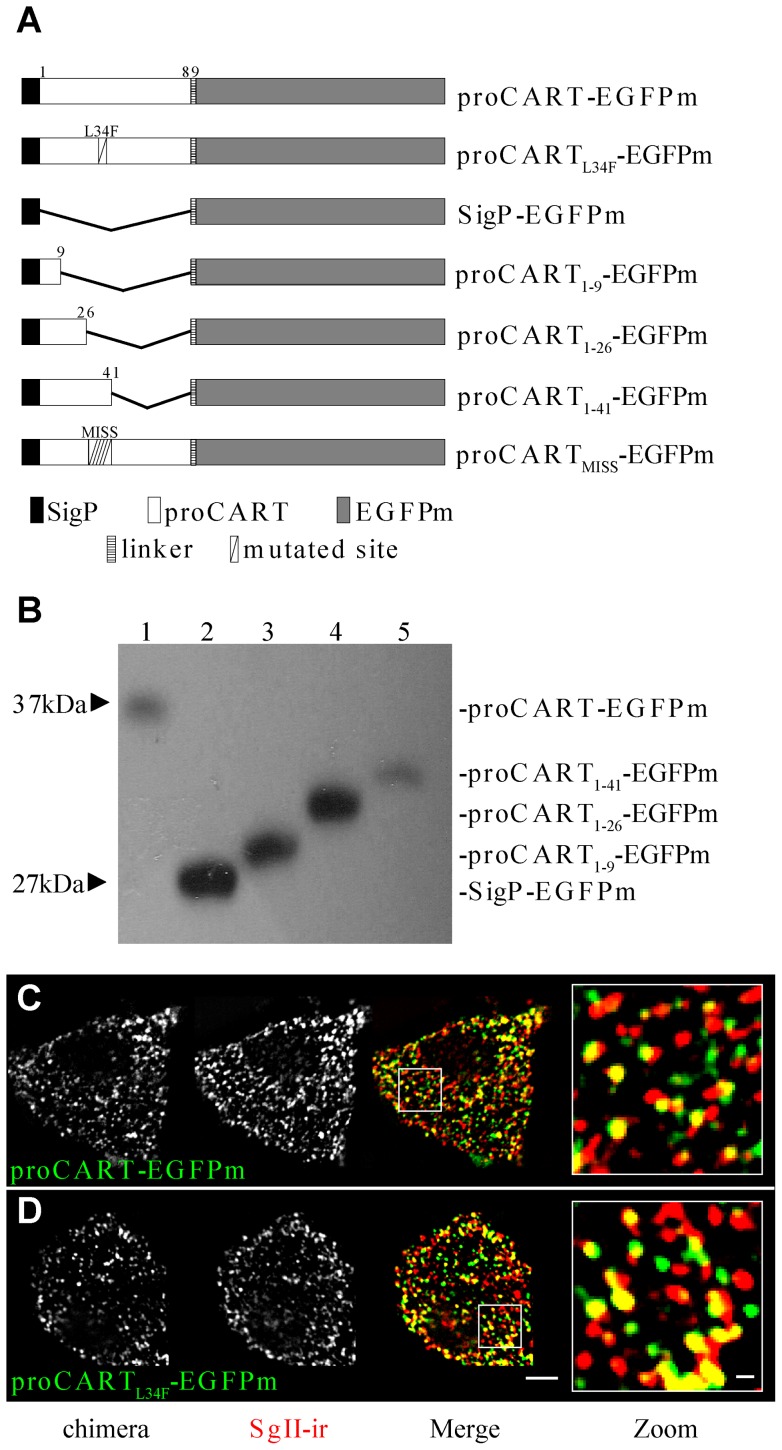
proCART-EGFPm and proCART_L34F_-EGFPm colocalize with SgII-ir. (A) Schema depicting all CART-EGFPm fusion proteins used in the study. Positions of amino acid residues from proCART_1–89_ fragments are indicated by the numbers alongside each fusion protein. (B) Fusion proteins were expressed in PC12 cells and cell lysates were analyzed by western blotting with anti-GFP antibody. Confocal images of PC12 cells transfected with proCART-EGFPm (C) or proCART_L34F_-EGFPm (D). Aldehyde-fixed cells were subjected to immunofluorescence protocols using SgII antibody. (C y D) Left panel: EGFPm signal. Middle: SgII-ir signal. Right panel: Merge of both signals. Zoom from merge image showing spots with both signals. Scale bar: 2 μm; zoom: 0.25 μm.

In order to dissect the proCART domain necessary to sort it to the regulated secretory pathway, we constructed several fusion proteins (Fig. 1A) fusing a monomeric form of EGFP (EGFPm) [16] to the carboxy-end of full-length proCART_1–89_ or to different fragments of proCART. All fusion proteins included the pre-proCART signal peptide (SigP), to enter the cellular secretory machinery. The selection of proCART_1–9_ and proCART_1–41_ fragments was based on described processing sites for proCART [13]. The proCART_1–26_ fragment was selected because two alternatively spliced forms of proCART are known to exist in rodents [1]. This alternative splicing described yields an isoform of proCART with an additional 13 amino acids inserted between positions 26–27.

As shown in Figure 1B, proCART-EGFPm migrated at the expected molecular mass of 37 kDa, corresponding to the molecular mass of proCART_1–89_ (10 kDA) and of EGFPm (27 kDA). Similarly, SigP-EGFPm, bearing only the SigP fused to EGFPm, showed a molecular mass of 27 kDa corresponding to EGFPm. Each fusion protein showed a single band at the estimated molecular mass indicating that the SigP was adequately recognized and removed (Fig. 1B).

The auto-fluorescent signal of proCART-EGFPm was distributed throughout the cytoplasm with a punctuate pattern (Fig. 1C). As expected, proCART-EGFPm autofluorescence colocalized with secretogranin II- immunoreactivity (SgII-ir), a marker of secretory granules, the hallmark of regulated secretory pathway (Fig. 1C). Remarkably, proCART_L34F_-EGFPm autofluorescence showed a punctate subcellular pattern indistinguishable from proCART-EGFPm and also colocalized with SgII-ir (Fig. 1D). Thus, in PC12 cells, the L34F mutation did not change the subcellular distribution pattern of proCART.

**Figure 2:**
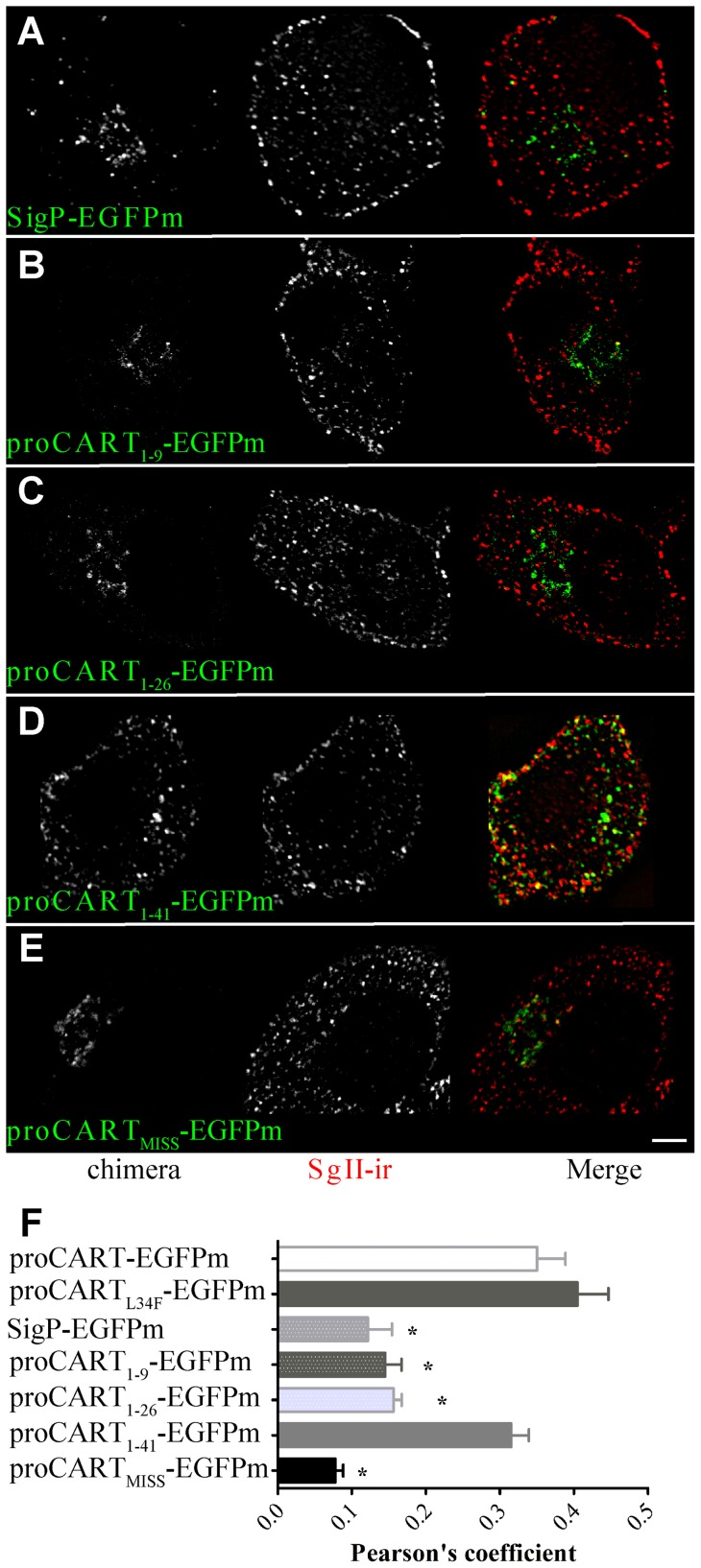
Subcellular distribution of CART-EGFPm fusion proteins. Confocal images of PC12 cells transfected with SigP-EGFPm (A), proCART_1–9_-EGFPm (B), proCART_1–26_-EGFPm (C), proCART_1–41_-EGFPm (D) or proCART_MISS_-EGFPm (E) expression vectors. Aldehyde-fixed cells were subjected to immunofluorescence protocols using SgII antibody. (A–E) Left panel: EGFPm signal. Middle: SgII-ir signal. Right panel: Merging of both signals. (F) Pearson's coefficient values for the colocalization of each of the fusion protein with SgII-ir (* P<0.001, One-way ANOVA followed by Dunnet's post-hoc test).

The autofluorescence of the fusion protein bearing only the signal peptide (SigP-EGFPm) yielded a perinuclear distribution, a location occupied by the Golgi apparatus complex (Fig. 2A and Fig. S1). This perinuclear pattern of distribution was replicated by proCART_1–9_-EGFPm (Fig. 2B) and proCART_1–26_-EGFPm (Fig. 2C). Interestingly, the fusion of the first 41 amino acids of proCART to EGFPm (proCART_1–41_-EGFPm) yielded a clear punctate pattern, homogenously distributed throughout the cytoplasm (Fig. 2D). Consistently, autofluorescence of SigP-EGFPm (Fig. 2A), proCART_1–9_-EGFPm (Fig. 2B) and proCART_1–26_-EGFPm (Fig. 2C) did not colocalize with SgII-ir. In contrast, proCART_1–41_-EGFPm showed robust colocalization with SgII-ir (Fig. 2D). Thus, proCART_1–41_-EGFPm (Fig. 2D) showed the same subcellular pattern than proCART-EGFPm (Fig. 1C) and proCART_L34F_-EGFPm (Fig. 1D).

Quantitative analysis of colocalization between the different fusion proteins and SgII-ir, confirmed that proCART-EGFPm, proCART_L34F_-EGFPm and proCART_1–41_-EGFPm have similar high levels of colocalization with SgII-ir (Fig. 2F). In contrast, SigP-EGFPm, proCART_1–9_-EGFPm and proCART_1–26_-EGFPm have significantly lower levels of colocalization with SgII-ir, compared to proCART-EGFPm (Fig. 2 F: * P<0.001; One-way ANOVA followed by Dunnet post-hoc test). Therefore, the proCART_1–41_ fragment is necessary and sufficient to reroute EGFPm towards the secretory granules.

### *In silico* modelling of proCART

**Figure 3:**
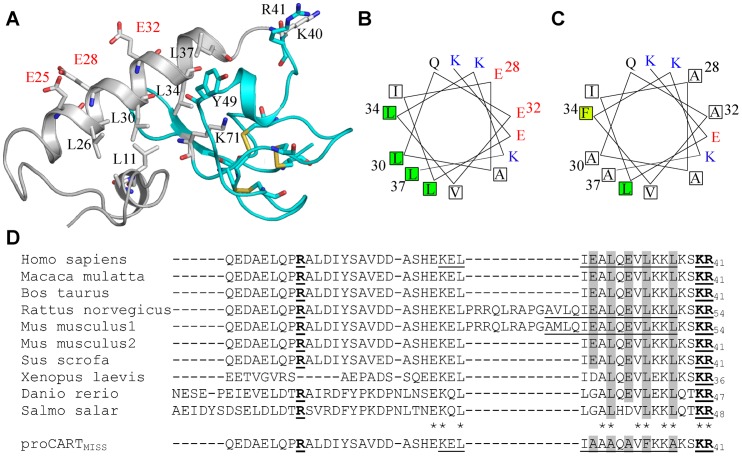
*In silico* modeling of proCART. (A) proCART was modeled starting from the NMR- derived CART_48–89_ structure (cyan). The modeling of the N-terminal region (gray) yielded an alpha-helix domain formed by residues 24–37. Disulfide bonds are indicated in yellow. (B) Helical wheel projection of proCART alpha-helix formed by residues 24–37. Leucines are indicated in green, glutamates in red and lysines in blue. (C) Helical wheel projection of proCART_MISS_. Substituted residues are shown in squares. Alanine substitution decreases the amphipathicity. (D) Alignment of proCART from several species. proCART alpha-helix structures predicted by NPS@ server (amino acids 24–37 for human, and 36–50 for rodent isoform are underlined. Residues modified in proCART_MISS_ are highlighted in gray. Asterisks show conserved residues among species. The basic sites involved in proCART processing are bolded and underlined.

Considering the significance role of the N-terminal domain in proCART subcellular localization, we generated an *in silico* model of proCART (Fig. 3A) starting from the published NMR-derived CART_48-89_ structure of its C-terminal region (cyan in Fig. 3A) [26]. The N-terminal domain of proCART presented a central alpha-helix of 14-residues which spans from amino acid 24 to 37 of proCART. The L34 residue together with L26, L30 and L37 formed a hydrophobic surface on the proCART alpha-helix On the contrary; residues E25, E28 and E32 are located on the opposite surface of the alpha-helix structure. The dibasic sites for the proteolitic cleavage of proCART are located at the loop formed by residues 38 to 44 (K40–R41) and in a small alpha-helix motif formed by residues 46 to 48 (K47–K48). The C-terminal part of the structure is identical to that obtained from NMR experiments, with 3 cysteine bridges stabilizing the folding. Therefore, the sorting signal of proCART towards the secretory granules contains an amphipathic alpha-helix structure in the N-terminal domain of proCART.

The projection of proCART alpha-helix into a wheel diagram also showed a clear amphipathicity of residues around the alpha-helix. The hydrophobic surface is formed by residues L26, L30, L34 and L37 and the polar surface is formed by residues K24, E25, E28, E32, K35 and K36 (Fig. 3B). An alignment of proCART from several species showed a high conservation of these residues (Fig. 3D). Interestingly, proCART from mouse and rat showed an additional alternatively spliced form, which adds 13 residues at the beginning of the predicted alpha helix within the residues 26 and 27 of proCART yielding a proCART long form [1]. Rat proCART short form sequence has not been submitted to NBCI data base. Thus, it was not included in the proCART species alingment shown in Figure 3D. However, the prediction of the secondary structure using NPS@ server [25] for rat and mouse proCART also yielded an alpha-helix structure coincident with human proCART_27–37_ sub-region (underlined, Fig. 3D). Thus, our results suggest that the highly conserved proCART alpha-helix core (proCART_27–37_) would be critical as a sorting signal.

There are two reported EST sequences supporting the existence of the spliced form of pre-proCART mRNA in rats (CB733430; CF977890). In addition, an EST sequence (EB366041) from guinea pig documents the existence of the pre-proCART mRNA long form in this rodent. In order to understand why rodent but not human proCART shows two alternative spliced forms, we compared the genomic sequences including the coding region of exon-1, intron-1 and exon-2 of mouse, rat and human (Table S2). This comparison showed that both spliced forms described in rat and mouse are due to the use of two alternative 3-ends of intron-1 (Table S2, both highlighted in gray). The analysis also showed a high degree of conservation between rodents and human of the weak polypyrimidine tracks associated to both 3′-end suggesting that both splice forms should be present in humans as well as in rodents. However, the evolutionary insertion of an extra nucleotide, 26 nucleotides downstream of the proximal 3′-end of human intron-1 (Table S2, adenosine in red), would change the open reading frame explaining the inexistence of the long proCART isoform in humans.

### Decrease in amphipathicity of the proCART alpha-helix causes its missorting

The replacement of leucine 34 for phenylalanine (proCART_L34F_-EGFPm) does not alter the hydrophobic nature of the alpha-helix hydrophobic surface. Consistently, this mutation did not change the subcellular localization of the fusion protein bearing the mutation in PC12 cells. Thus, we evaluated whether the amphipathicity of the alpha-helix is critical for proCART sorting, as it has been previously shown for other regulated secretory proteins [37]. To challenge this idea, we substituted the residues L30, L37, E28, and E32 for alanines – in addition to L34F- to decrease the amphipathicity of the alpha-helix in proCART (Fig. 2C). This new fusion protein was named proCART_MISS_-EGFPm. As can be seen in Figure 2E, proCART_MISS_-EGFPm showed a clear perinuclear pattern when it was expressed in PC12 cells. Consistently, the colocalization analysis with SgII showed a significant reduction of the colocalization of proCART_MISS_-EGFPm compared to proCART-EGFPm (Fig. 2F). Thus, the amphipathicity reduction in proCART alpha-helix significantly changed the subcellular localization of proCART.

**Figure 4:**
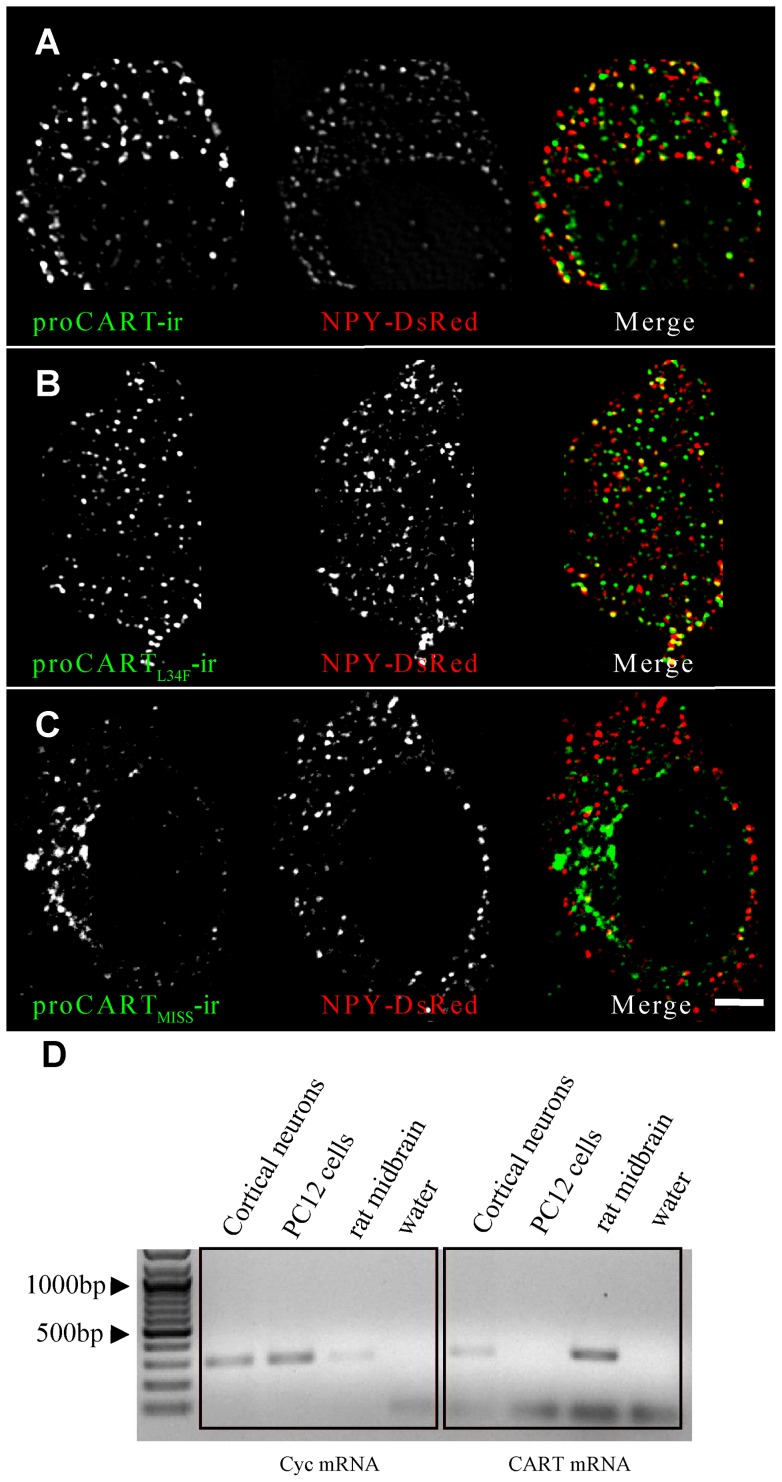
proCART and proCART_L34F_, but not proCART_MISS_ colocalized with NPY-DsRed. proCART species without EGFP were cotransfected in PC12 cells with NPY-DsREd expressing vector and detected with an antiCART specific polyclonal rabbit antibody. (A) proCART and (B) proCART_L34F_ showed a punctate pattern and both colocalized with NPY-DsRed. However, (C) proCART_MISS_ showed a perinuclear pattern and did not colocalize with NPY-DsRed. (D) RT-PCR for CART mRNA in rat primary cortical neurons, PC12 cells and rat midbrain at the level of the Edinger-Westphal nucleus. Total RNA was extracted with Trizol and 0.5 µg of each RNA was used for each RT-PCR. A set of specific primers (listed in Table S1) to amplify a 322 bp fragment of pre-proCART mRNA were used. Cyclophilin (Cyc) mRNA was also amplified as a control.

To further rule out that the sorting behavior of the fusion proteins could be due to artifacts of using EGFPm, we analyzed the sorting of proCART, proCART_L34F_ and proCART_MISS_ species without EGFP using a specific anti-CART antibody [38]. It has been reported the natural expression of proCART mRNA in PC12 cells [39]. However, PC12 cells used in this report did not show detectable levels of proCART mRNA (Fig. 4D). Immunoreactivity for proCART and proCARTL34F overexpressed in PC12 cells presented the same punctate subcellular pattern (Fig. 4A, B). However, the immunoreactivity of proCART_MISS_ showed a perinuclear subcellular pattern (Fig. 4C). Co-expression with NPY-DsRed, a validated secretory granule marker [40], and colocalization analyses showed that proCART and proCART_L34F_ have the same level of colocalization with NPY-DsRed. Nevertheless, proCART_MISS_ had significantly lower levels of colocalization with NPY-DsRed. Pearson's coefficient values for the colocalizations with NPY-DsRed were 0.31±0.02; 0.33±0.03 and 0.20±0.02* for proCART, proCART_L34F_ and proCART_MISS_, respectively (*P<0.01; One-way ANOVA followed by Dunnet post-hoc test). The prediction of the secondary structure using NPS@ server [25] and also by i*n silico* molecular modeling showed that proCART_MISS_ conserved the predicted alpha helix structure (data not shown). Additionally, the modification of the hydrophobic surface but not of the polar surface of predicted proCART alpha helix significantly lowered the granular localization of proCART (Fig. S2).

### proCART_MISS_ showed high basal secretion levels and resistance to barium stimulation

**Figure 5:**
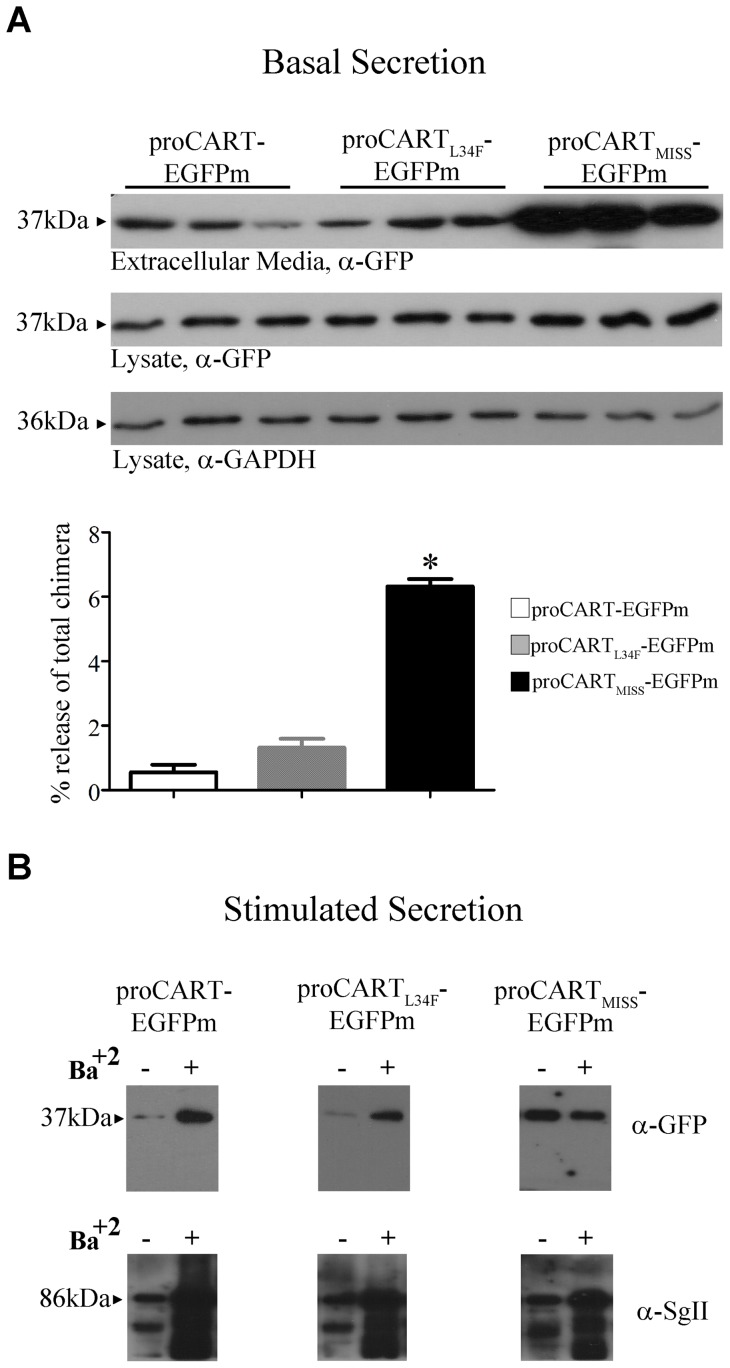
proCART-EGFPm and proCART_L34F_-EGFPm are released upon barium stimulation, and proCART_MISS_-EGFPm is secreted constitutively. (A) Basal secretion. The 48 h incubation media of 3 independent samples for proCART-EGFPm, proCART_L34F_-EGFPm and proCART_MISS_-EGFPm were immunoblotted with an anti-GFP antibody to determine the secretion in the absence of stimulus. Twenty μg of each cell lysate were immunoblotted with anti-GFP and anti-GAPDH antibodies. Secreted levels were plot as percentage of fusion protein released/total fusion protein expressed. (*P<0.0001; One-way ANOVA followed by Dunnet post-hoc test). (B) Stimulated secretion. PC12 cells transfected with either proCART-EGFPm, proCART_L34F_-EGFPm or proCART_MISS_-EGFPm and 48 h after, cells were incubated for 30 min in basal medium (− lanes) and thereafter for additional 30 min in 2 mM Ba^2+^-medium (+ lanes). proCART-EGFPm, proCART_L34F_-EGFPm and proCART_MISS_-EGFPm secreted in the respective media were analyzed by western blotting with anti-GFP antibody. In all cases immunoblots were also revealed with anti-SgII antibody to control for the presence of the normal response to barium-stimulation.

To further prove the significance of the amphipathic helix of CART in appropriate sorting, we studied the capacity of the CART-EGFPm fusion proteins to be secreted constitutively or in a stimulated fashion. In basal conditions the extracellular media of PC12 cells transfected with proCART_MISS_-EGFPm was enriched 6 times with the recombinant protein compared to the extracellular media of PC12 cells transfected with proCART-EGFPm (Fig. 5A). As expected, based on similar effects of proCART_L34F_-EGFPm and proCART-EGFPm, they presented similar low constitutive secretion levels. In contrast, proCART_MISS_-EGFPm showed a significant higher constitutive secretion that is consistent with its lack of colocalization with SgII-ir (Fig. 2F).

To study the stimulated secretion from secretory granules, PC12 cells transfected with the different CART-EGFPm fusion proteins were exposed to barium, a potent exocytotic secretagogue [41]–[43]. As can be seen in Figure 5B, the stimulation with 2 mM barium increased extracellular levels of proCART-EGFPm and proCART_L34F_-EGFPm, compared to their respective controls in basal conditions. In contrast, proCART_MISS_-EGFPm was insensible to barium-induced stimulation (Fig. 5B). To control barium stimulatory capacity in all cases, barium-induced secretion of SgII was measured from the same samples. In all cases, barium induced the secretion of an 86 kDa band corresponding to SgII (Fig. 5B). Thus, the amphipathicity of the alpha-helix is necessary for the increase in proCART secretion induced by barium stimulation.

## Discussion

Here we report that an amphipathic alpha-helix present in the N-terminal region of proCART is necessary for the sorting of this propeptide to secretory granules in the PC12 cell line, which does not express prohormone converstases PC1/3 and PC2. Thus, our results indicate that proCART does not depend on PCs-dependent cleavage to readily access the regulated secretory pathway. We also report that the L34F mutation in proCART that has been associated to familiar obesity [14] does not affect the appropriate sorting of proCART in PC12 cells. Interestingly, by reducing the amphipathic feature of the alpha-helix changing 2 leucines for alanines in the hydrophobic surface and two glutamate for alanine in the hydrophilic surface prevented the correct sorting of proCART, redirecting it to the constitutive secretory pathway. Furthermore, the replacement of only two leucines by alanine in the hydrophobic surface of proCART alpha helix is sufficient to decrease its sorting to the secretory granules. The critical role of the proCART amphipathic alpha-helix in its sorting is documented by data obtained with two independent strategies. First, only those CART-EGFPm fusion proteins harboring the amphipathic alpha-helix colocalized with endogenous SgII-like immunoreactivity. Second, CART-EGFPm fusion proteins presenting a punctate subcellular pattern and colocalizing significantly with SgII-ir, were readily released upon exocytotic stimulation. In contrast, CART-EGFPm fusion proteins presenting a perinuclear pattern are secreted through the constitutive pathway.

Positive correlation between subcellular localization (punctate pattern) and regulated secretion have been used to show the sorting through the regulated pathway of the granins [41], [44]. In our study, CART-EGFPm fusion proteins with an intact amphipathic alpha-helix and presenting punctate subcellular pattern were readily released upon stimulation, further proving their sorting through the regulated secretory pathway. In this sense it is not surprising that proCART_L34F_ bearing a conservative mutation on the hydrophobic surface of the predicted alpha-helix is also readily sorted to the regulated secretory pathway and released from transfected PC12 cells upon depolarizing stimulation.

Even though EGFP has been extensively used as a fluorescent reporter in sorting studies, caution must be taken because it has been shown that EGFP oligomerizes inside the secretory pathway lumen [45], [46]. This oligomerization is sufficient to facilitate the entrance of EGFP into secretory granules [45]–[48]. Thus, we used EGFPm (EGFP_A206K_) that has been shown to reduce its oligomerization [14] and methodological artifacts due to EGFP oligomerization [49]. The possibility that the observed sorting of CART-EGFPm fusion proteins could be due to artifacts of using EGFPm was further ruled out analyzing the sorting of proCART, proCART_L34F_ and proCART_MISS_ species without EGFPm, using a CART specific antibody [38] and their colocalization with NPY-DsRed, a validated secretory granule marker [40].

Interestingly, we observed that several, but not all the spots in a cell showed colocalization (Fig. 1C). The spots without EGFPm signal and positive for SgII-ir could be explained by the presence of old granules. However, the spots with EGFPm signal and without SgII-ir are paradoxical. A closer look at the labeled spots in our study shows different proportions of each signal by spot, suggesting that the content of each secretory granule is not equal and that their composition appears not to be constant. This variability in content has been previously described for chromogranin A and B content of PC12 secretory granules [7]. Furthermore, it has been suggested that segregation among secretory granules could be explained by the different aggregative properties of each cargo protein in the lumen of the regulated secretory pathway [49].

Two mechanisms have been proposed to explain the role of alpha-helix structures in the sorting of proteins towards the regulated secretory pathway. First, it has been reported that proteins such as PC1/3, PC2 and carboxypeptidase E (CPE) are sorted to the secretory granules due to the presence of cis-alpha-helix domains that anchor them to membranes allowing their sorting [11], [37], [50], [51]. In addition, it has been shown that these proteolytic enzymes are responsible for the sorting of propeptides such as prorenin [52] and proneurotensin [53] through their dibasic sites. Dikeakos and Reudelhuber [54] have thoroughly reviewed the evidence proposing that PCs and CPE could work as receptors facilitating the sorting of neuropeptide precursors. proCART has several basic sites that are susceptible to proteolytic processing by PCs [10], [13]. Thus, it could be possible that proCART sorting depends on interactions with PCs. However, we have shown that proCART is readily sorted in PC12 cells that do not express PCs. We cannot rule out that the interactions between PCs and proCART could facilitate its sorting in other cell contexts. Second, it has been shown that some proteins can be sorted to the secretory granules through amphipathic alpha-helices that allow their self-aggregation [41]. For instance, SgII has an alpha-helix conformation in its N-terminal 25–41 fragment that is necessary and sufficient to be sorted towards the regulated secretory pathway [41]. Another example is the neuropeptide precursor, pro-somatostatin (proSST). In this case, it has been shown that decreasing the hydrophobic nature of the hydrophobic surface, by replacing two leucines by alanines, is sufficient to abolish proSST sorting towards the regulated secretory pathway [55]. It has been shown that some proteins that are sorted through this mechanism, such as chromogranins, are granulogenic [56]. Interestingly, the amphipathic alpha-helix of proCART, identified herein as its sorting domain, has similar highly conserved characteristics to both proSST and SgII sorting domains. It is worth mentioning that proCART was originally isolated and sequenced as proSST-like polypeptide [57], and a similar sorting mechanism perhaps would emphasize the common evolutionary origin of both peptides. According to NPS@ server, proCART_MISS_ preserves the alpha-helix structure, but loses the amphipathicity. Thus, the amphipathicity of proCART alpha-helix must be responsible for its sorting. It has been shown that the hydrophobic surface of the amphipathic alpha-helices is the major contributor to the sorting towards the regulated secretory pathway [37]. Our results showing that proCART is missorted if only two leucines of the hydrophobic surface of proCART alpha-helix are replaced by alanine further support the critical role of hydrophobic patches in alpha-helices as sorting determinants. It is tempting to suggest that this amphipathic alpha-helix allows proCART to be sorted towards secretory granules by a granulogenic-like mechanism, such as reported by Beuret et al. [56] for other regulated cargo proteins. This is further supported by the observation of a heterogenic pool of secretory granules containing proCART and SgII in each PC12 cell.

In rodents there is strong evidence about the existence of a long proCART isoform. The incidence in the proCART sorting of the additional 13 amino acids in long proCART isoform was not studied in this report. However, the *in silico* analysis showed that this insertion does not affect the alpha-helix core structure (Fig. 3D). Thus, we think that the 13 additional amino acids would not affect the proCART sorting, but we cannot discard it. On another hand, we propose a hypothesis to explain the inexistence of the long proCART isoform in humans (Table S2). The alignment analysis showed an additional nucleotide between both 3′-ends of intron-1 in human pre-proCART gene that it is absent in mouse and rat. This additional nucleotide would change the proCART ORF forming a premature stop codon in this eventual alternative human pre-proCART mRNA leading this incipient transcript to the nonsense-mediated mRNA decay pathway. Further studies are necessary for test this hypothesis.

proCART_L34F_ behaves similarly to proCART in PC12 cells. The helical wheel projection showed that L34 is located in the hydrophobic surface of proCART amphipathic alpha-helix. The alignment of proCART of different species shows a high conservation of the L34 residue supporting a conserved function. The change of the Leucine in residue 34 to Phenyalanine does not modify the hydrophobic surface of proCART alpha-helix and therefore, it should not affect proCART sorting, as observed in our study. Our results differ from the previous study of Yanik et al. [15] in which it was suggested that the L34F mutation would missort proCART. These authors studied the sorting of proCART_L34F_ in AtT20 cells that express PC1/3, which recognizes and cleaves the dibasic site K_40_R_41_ in proCART to generate the CART_42–89_ peptide [10]. Thus, in AtT20, it should be expected secretion of CART_42–89_ peptide upon stimulus. However, when proCART_L34F_ was expressed in AtT20 cells, the levels of CART_42–89_ peptide were dramatically decreased [15]. In contrast, the PC12 cells used in our study do not express PC1/3 and PC2 (Fig. S3), as previously described [58]. Thus, in our system proCART sorting was isolated from its processing. At present, we do not have an explanation for the different behavior of proCART_L34F_ in AtT20 cells [15] versus PC12 cells (our study). Both cell lines are endocrine-like cells and have been extensively used to study the sorting of secreted proteins and peptides [54]. The possibility that the decrease of CART peptide levels derived from proCART_L34F_ in AtT20 cells could be due to a poorer processing of proCART_L34F_ should be further explored. However, the differences in proteolytic activity between Att20 and PC12 cells do not explain in its own the differential sorting behavior of the proCART_L34F_ observed in both cellular contexts.

In conclusion, we have identified a proCART sorting domain necessary and sufficient to enter the regulated secretory pathway. This domain contains a predicted alpha-helix whose amphipathic character is essential for proCART sorting. The L34F mutation associated to human obesity is located in this proCART alpha-helix [14]. However, this mutation is not sufficient to missort proCART in PC12 cells. The possibility that the changes in CART plasma levels observed in carriers of the L34F mutant form of proCART is due to a failure in proCART processing, should be further explored.

## Acknowledgments

The authors thank the generous donations of: 1) CART antibody from Dr. Michael J. Kuhar (Emory University, Atlanta, USA); 2) human proCART cDNA from Dr. Patrick Keller (University Medical Center, Geneva, Switzerland); 3) NPY-DsRed expression vector from Dr. Xu (Institute of Biophysics, Chinese Academy of Sciences, Beijing, China).

## Supporting Information

Figure S1 **SigP-EGFPm is accumulated in the TGN of PC12 cells.** (A) proCART-EGFPm autofluorescence showed a granular subcellular pattern and TGN38 immunoreactivity(TGN38-ir), a trans-Golgi marker, showed the typical perinuclear subcellular pattern. (B) SigP-EGFPm fusion protein autoflorescence showed similar subcellular pattern than TGN38-ir. Pearson values were 0.12±0.01 (8 cells; 208 images) for proCART-EGFPm vs TGN38-ir and 0.21±0.02 (8 cells; 208 images) for SigP-EGFPm vs TGN38-ir. TGN38-ir was obtained with Anti-TGN38 monoclonal antibody (1∶1000; MA3-063; Pierce Biotechnology). Scale bar: 2 μm.
(TIF)

Figure S2 **Leucines 30 and 37 are necessary for adequate proCART subcellular localization.** Considering the amphipathicity of proCART alpha helix domain, we generated two additional novel mutated CART-EGFPm fusion proteins: 1) Replacement by alanines of residues E28 and E32 from the polar surface and 2) Replacement by alanines of residues L30 and L37 from the hydrophobic surface. A) proCART-EGFPm autofluorescence showed a granular subcellular pattern colocalizing with SgII-ir. B) proCART_EE.AA_-EGFPm autofluorescence showed the same subcellular pattern than proCART-EGFPm. C) proCART_LL.AA_-EGFPm showed a significantly lower colocalization with SgII-ir. Pearson values for colocalization with SgII-ir were: 0.29±0.02 (4 cells) for proCART-EGFPm, 0.28±0.03 (4 cells) for proCART_EE.AA_-EGFPm and 0.12±0.02 (4 cells) for proCART_LL.AA_-EGFPm. Scale bar: 2 μm.
(TIF)

Figure S3 **PC12 cells do not express promohormone convertases PC1/3 and PC2.** RT-PCR showed the absence of PC1/3 and PC2 expression in PC12 cells. However, PC12 cells express carboxipeptidase E (CPE). Cyclophilin was amplified as a re-trotranscription control. cDNA from lateral hypothalamus (C+) was used as a positive control for each PCR. Total RNA was extracted by the Trizol method and 0.5 μg of total RNA was used for each retro-transcription, and 1 μL of cDNA was used in each PCR reaction. The PCR program was 94°C×10 min; 30 cycles (94°C×30 seg, 55°C×30 seg, 72°C×30 seg); and 72°C 10 min. Primers used for RT-PCR are listed in table S1.
(TIF)

Table S1 **List of primers used in RT-PCR.**
(DOC)

Table S2 **Nucleotidic analysis of pre-proCART gene intron-1. Posible explanation for the absence of a human long proCART isoform.** The pre-proCART gene sequences corresponding to the coding region of exon-1, intron-1 and exon-2 for the three species were aligned and analyzed. The 5′-end of intron-1 is highlighted in black. The 3′-ends of the intron yielding the longer proCART isoform documented in rodents are highlighted in gray (proximal 3′-end). The 3′-ends of the intron yielding the shorter isoform documented in both rodents and humans are highlighted in dark gray (distal 3′-end). Both 3′-end splicing sites are very similar and have weak polypyrimidine tracks, yielding similar probability to be a 3′-end splicing site (Lopez, A. J.; Alternative splicing of pre-mRNA: developmental consequences and mechanisms of regulation. *Annu. Rev. Gen.*, *32*, 279–305, 1998). Thus, the lack of the long proCART isoform in human, most probably is not due to differences in the rate of splicing between both 3′-ends. Interestingly, the presence of an additional nucleotide between both 3′-ends of the human intron-1 sequence (adenosine depicted in red). If the human proximal 3′-end is used the additional adenosine changes the open reading frame leading to a premature stop codon (codon TAA in human exon-2). We think that this premature stop codon would explain the lack of the long proCART isoform in humans. It is worth to mention that in Douglass et al. (1995), it was reported that 2/3 of the clones they obtained from rat mRNA corresponded to the short proCART isoform. However, the physiological consequences of the presence of both proCART isoforms in rodents are presently unknown.
(DOC)
