# Supplementary figures and images for: An Amphipathic Alpha-Helix in the Prodomain of Cocaine and Amphetamine Regulated Transcript Peptide Precursor Serves as Its Sorting Signal to the Regulated Secretory Pathway
Source: PLoS One. 2013 Mar 19;8(3):e59695. doi: 10.1371/journal.pone.0059695 (PMC3602189; doi:10.1371/journal.pone.0059695)

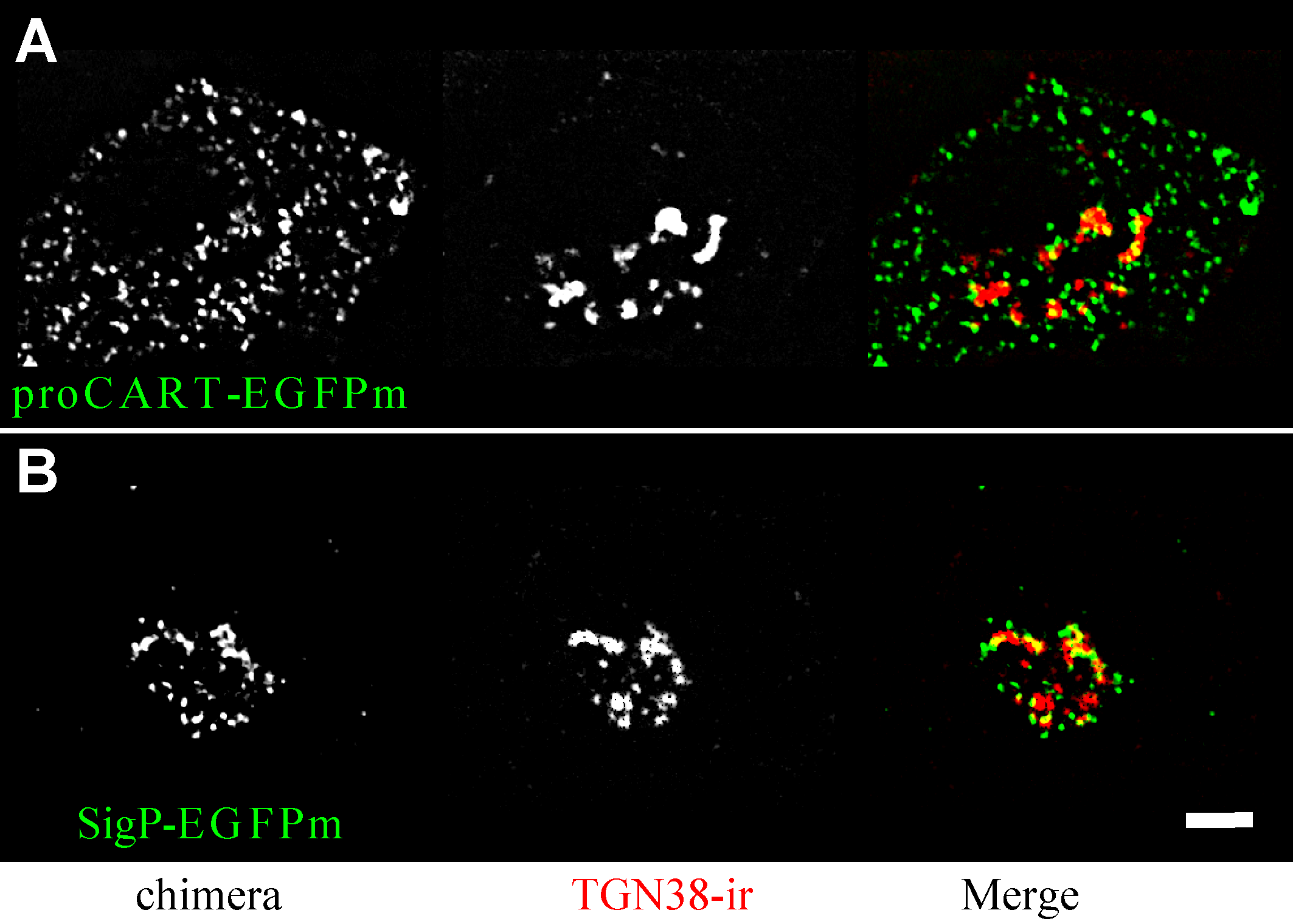

Supplement: Figure S1 — SigP-EGFPm is accumulated in the TGN of PC12 cells. (A) proCART-EGFPm autofluorescence showed a granular subcellular pattern and TGN38 immunoreactivity(TGN38-ir), a trans-Golgi marker, showed the typical perinuclear subcellular pattern. (B) SigP-EGFPm fusion protein autoflorescence showed similar subcellular pattern than TGN38-ir. Pearson values were 0.12±0.01 (8 cells; 208 images) for proCART-EGFPm vs TGN38-ir and 0.21±0.02 (8 cells; 208 images) for SigP-EGFPm vs TGN38-ir. TGN38-ir was obtained with Anti-TGN38 monoclonal antibody (1∶1000; MA3-063; Pierce Biotechnology). Scale bar: 2 μm. (TIF) [file pone.0059695.s001.tif]

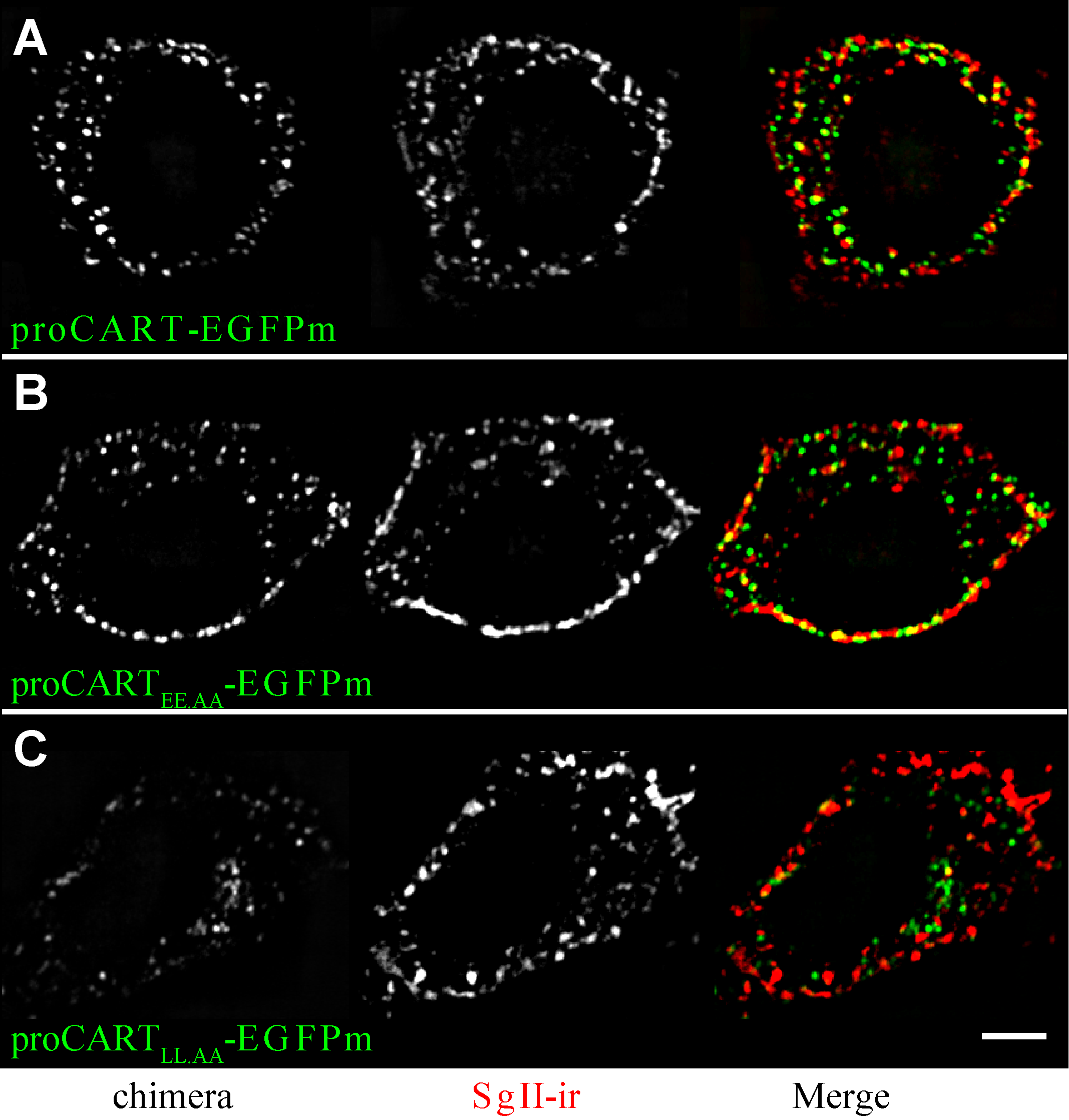

Supplement: Figure S2 — Leucines 30 and 37 are necessary for adequate proCART subcellular localization. Considering the amphipathicity of proCART alpha helix domain, we generated two additional novel mutated CART-EGFPm fusion proteins: 1) Replacement by alanines of residues E28 and E32 from the polar surface and 2) Replacement by alanines of residues L30 and L37 from the hydrophobic surface. A) proCART-EGFPm autofluorescence showed a granular subcellular pattern colocalizing with SgII-ir. B) proCARTEE.AA-EGFPm autofluorescence showed the same subcellular pattern than proCART-EGFPm. C) proCARTLL.AA-EGFPm showed a significantly lower colocalization with SgII-ir. Pearson values for colocalization with SgII-ir were: 0.29±0.02 (4 cells) for proCART-EGFPm, 0.28±0.03 (4 cells) for proCARTEE.AA-EGFPm and 0.12±0.02 (4 cells) for proCARTLL.AA-EGFPm. Scale bar: 2 μm. (TIF) [file pone.0059695.s002.tif]

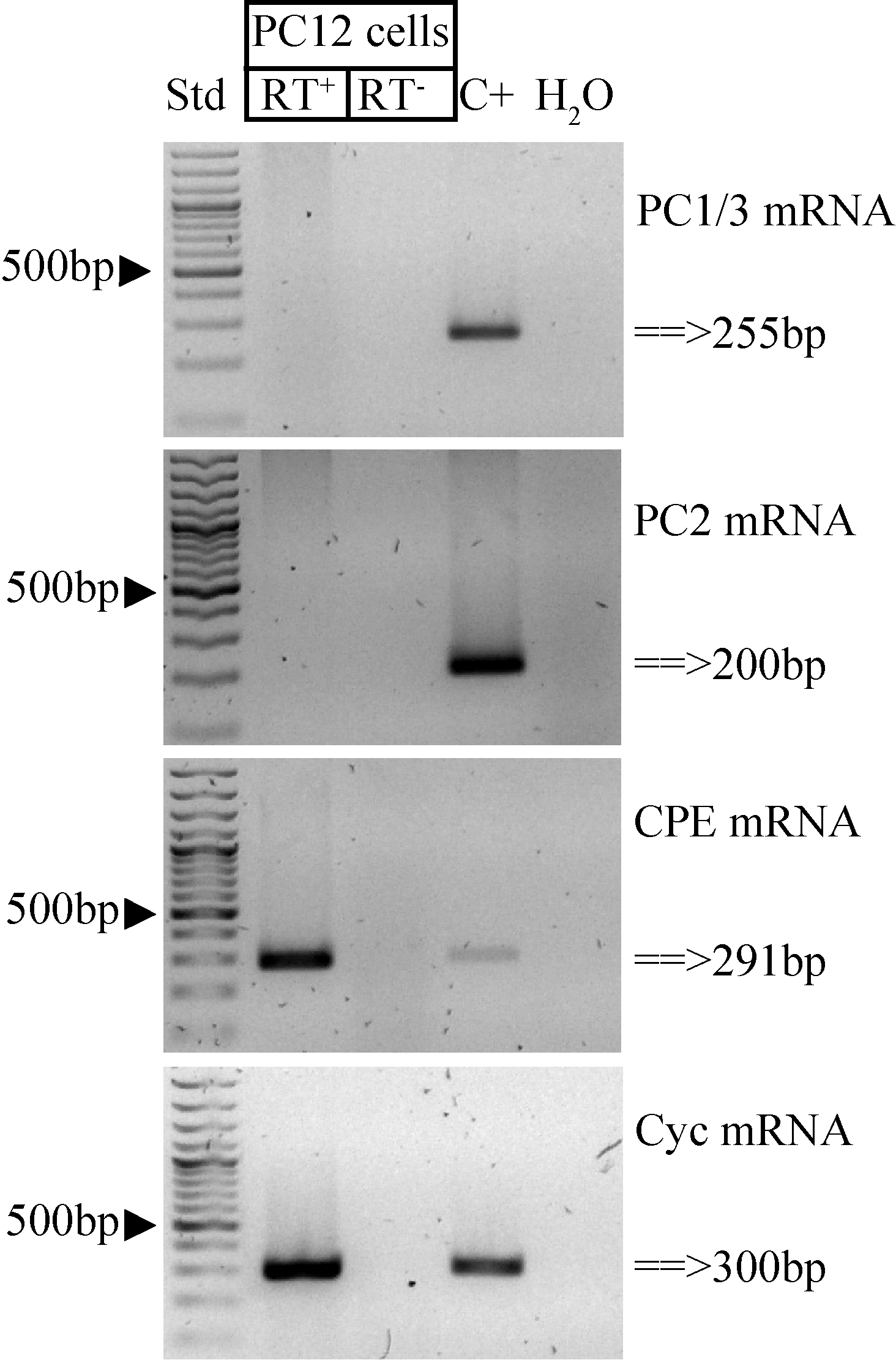

Supplement: Figure S3 — PC12 cells do not express promohormone convertases PC1/3 and PC2. RT-PCR showed the absence of PC1/3 and PC2 expression in PC12 cells. However, PC12 cells express carboxipeptidase E (CPE). Cyclophilin was amplified as a re-trotranscription control. cDNA from lateral hypothalamus (C+) was used as a positive control for each PCR. Total RNA was extracted by the Trizol method and 0.5 μg of total RNA was used for each retro-transcription, and 1 μL of cDNA was used in each PCR reaction. The PCR program was 94°C×10 min; 30 cycles (94°C×30 seg, 55°C×30 seg, 72°C×30 seg); and 72°C 10 min. Primers used for RT-PCR are listed in table S1. (TIF) [file pone.0059695.s003.tif]
