# Supplementary material for: An Amphipathic Alpha-Helix in the Prodomain of Cocaine and Amphetamine Regulated Transcript Peptide Precursor Serves as Its Sorting Signal to the Regulated Secretory Pathway
Source: PLoS One. 2013 Mar 19;8(3):e59695. doi: 10.1371/journal.pone.0059695 (PMC3602189; doi:10.1371/journal.pone.0059695)
Supplement: Table S1 — List of primers used in RT-PCR. (DOC) [file pone.0059695.s004.doc]

| Target | Primer sequence | Amplicon size |
| --- | --- | --- |
| CART rat  NM017110.1 | F:GCG CTG CAG GAA GTC CTG AAG  R:GAA GCA GCA GGG AAA GAG CCC | 322bp |
| PC1/3 rat  NM017091.2 | F:AAT CCT GTA GGC ACC TGG AC  R:GGA GTT TTT GGG TAC CAG GA | 255bp |
| PC2 rat  NM012746.1 | F:GAG AGG AGA CCT GAA CAT CA  R:GCA AGC CCT TCT GTG GTG CA | 200bp |
| CPE rat  NM013128.1 | F:GGG TTT GTC CGT GAC CTT CA  R:CAA AGT CTC TGA CAT CAT TTT CCA | 291bp |
| Cyc rat  NM017101.1 | F:CGT GCT CTG AGC ACT GGG GAG AAA  R:CAT GCC TTC TTT CAC CTT CCC AAA GAC | 300bp |
