# Supplementary material for: An Amphipathic Alpha-Helix in the Prodomain of Cocaine and Amphetamine Regulated Transcript Peptide Precursor Serves as Its Sorting Signal to the Regulated Secretory Pathway
Source: PLoS One. 2013 Mar 19;8(3):e59695. doi: 10.1371/journal.pone.0059695 (PMC3602189; doi:10.1371/journal.pone.0059695)
Supplement: Table S2 — Nucleotidic analysis of pre-proCART gene intron-1. Posible explanation for the absence of a human long proCART isoform. The pre-proCART gene sequences corresponding to the coding region of exon-1, intron-1 and exon-2 for the three species were aligned and analyzed. The 5′-end of intron-1 is highlighted in black. The 3′-ends of the intron yielding the longer proCART isoform documented in rodents are highlighted in gray (proximal 3′-end). The 3′-ends of the intron yielding the shorter isoform documented in both rodents and humans are highlighted in dark gray (distal 3′-end). Both 3′-end splicing sites are very similar and have weak polypyrimidine tracks, yielding similar probability to be a 3′-end splicing site (Lopez, A. J.; Alternative splicing of pre-mRNA: developmental consequences and mechanisms of regulation. Annu. Rev. Gen., 32, 279–305, 1998). Thus, the lack of the long proCART isoform in human, most probably is not due to differences in the rate of splicing between both 3′-ends. Interestingly, the presence of an additional nucleotide between both 3′-ends of the human intron-1 sequence (adenosine depicted in red). If the human proximal 3′-end is used the additional adenosine changes the open reading frame leading to a premature stop codon (codon TAA in human exon-2). We think that this premature stop codon would explain the lack of the long proCART isoform in humans. It is worth to mention that in Douglass et al. (1995), it was reported that 2/3 of the clones they obtained from rat mRNA corresponded to the short proCART isoform. However, the physiological consequences of the presence of both proCART isoforms in rodents are presently unknown. (DOC) [file pone.0059695.s005.doc]

**Mouse ATGGAGAGCTCCCGCCTGCGGCTGCTACCCCTCCTGGGCGCCGCCCTGCTGCTACTGCTA 60**

Rat ATGGAGAGCTCCCGCCTGCGGCTGCTACCCGTCCTGGGCGCCGCCCTACTGCTGCTGCTA 60

Human ATGGAGAGCTCCCGCGTGAGGCTGCTGCCCCTCCTGGGCGCCGCCCTGCTGCTGATGCTA 60

*************** ** ******* *** **************** ***** *****

Mouse CCTTTGCTGGGTGCCCGTGCCCAGGAGGACGCCGAGCTGCAGCCCCGAGCCCTGGACATC 120

Rat CCTTTGCTGGGTGCCGGTGCCCAGGAGGATGCCGAGCTGCAGCCCCGAGCCCTGGACATC 120

Human CCTCTGTTGGGTACCCGTGCCCAGGAGGACGCCGAGCTCCAGCCCCGAGCCCTGGACATC 120

*** ** ***** ** ************* ******** *********************

5’ -end

Mouse TACTCTGCCGTGGATGATGCGTCCCACGAGAAGGAGCTGgtcagtatttccctcgcctcg 180

Rat TACTCTGCCGTGGATGATGCGTCCCATGAGAAGGAGCTGgtcagtatttccctcgcctcg 180

Human TACTCTGCCGTGGATGATGCCTCCCACGAGAAGGAGCTGgtcggtattcccctcgctctc 180

******************** ***** *************** ***** *******

Mouse ggttcccccactggactaccacagggtccttgct-ttgcgtgcgtccccct-----ctat 234

Rat ggtttgcc--ctgggctgccacgggacccttgct-ttgcgtgcgtccctct-----ccac 232

Human gaccccct---tgagctgtcgccttgtctcttctcttgcacgcctccctcctccccccac 237

* * ** ** * * * * ** **** ** **** * * *

Mouse ccc-agtcccatcccagaaggcagagcccctgacgctggattaaaaaaaaaaaagtaccc 293

Rat ccc-agtcccatcccagaaggcagagaccctgac--tggatttaaaaaaaaatagt---c 286

Human ccccactcctattcccagagtcagggcgcggggagctgagcgcaacg-----------cc 286

*** * *** ** ** ** *** * * * ** ** *

Mouse tgggtaccctctaccagacaaaga-tattt-gagttcacgggctgcgggctgtctgtcga 351

Rat tgggtaccctctgccagacaaaga-tattt-gagttcatgggctgcgggctgcctgtcga 344

Human caggcacccactgccatccgaagagcgtctcgagctcacgggctcctggcagtctgttga 346

** **** ** *** * **** * * *** *** ***** * *** * **** **

Mouse gcgaatccctagttgtggccggtctgggcagtgtgctccttggaggctctggaaggtgag 411

Rat gcgaatccctagttgtggccagtctgggcactgtgcgccttgaaggctctagaagatgag 404

Human gcgaatccctcatcccggcccctctgagcaacagggaccccagcggctcagagacccgcg 406

********** * **** **** *** * ** ***** * * *

Mouse gtcaggtctagggactttgcccgcctggcttcctttccaccagtatctactcccctttgt 471

Rat gtcaggactagggactttgcctgccagacttcctctccaccagtatcttctcccctttgt 464

Human gtcagtacctgggacagcgtccgctaagtttccacccctcgaccatt-----ccctgtgt 461

***** * ***** * * ** **** ** * * ** **** ***

Mouse cccccgagtcctgctgccccgcggttgtgtgtcccgggctccttataact-gggctgaaa 530

Rat cccccgagtcccgctaccccgcggttgtatttcccgggctccctataact-gggctgaaa 523

Human ccgcggagtcccaccgcagagtgcgtgtgggtccggggctccttataactagggctggaa 521

** * ****** * * * * *** *** ******* ******* ****** **

Proximal 3’ -end

Mouse gtgagcacctgcgctgggctcgcagccaaggcggcaacttcgggctcccg-gcgctatgt 589

Rat gtgagcacctgcgctgggctcgcagccaaggcggcaacttcgggctcccg-gcgctgtgt 582

Human gtgcgcacctgggctgggctcgcagccaaggcggcaacttcaggctccga**a**gcggtgtgt 581

*** ******* ***************************** ****** *** * ***

Distal 3’ -end

Mouse **tgcagATCGAAGCGTTGCAAGAAGTCCTGAAGAAGCTCAAGAGTAAACGCATTCCGATCT** 649

Rat tgcagATTGAAGCGCTGCAGGAAGTCCTGAAGAAGCTCAAGAGTAAACGCATTCCGATCT 642

Human tgcagATCGAAGCGCTGCAAGAAGTCTTGAAGAAGCTCAAGAGTAAACGTGTTCCCATCT 641

******* ****** **** ****** ********************** **** ****

Mouse ACGAGAAGAAGTACGGCCAAGTCCCCATG 678

Rat ATGAGAAGAAGTACGGCCAAGTCCCCATG 671

Human ATGAGAAGAAGTATGGCCAAGTCCCCATG 670

* *********** ***************
